# At the Intersection of Natural Structural Coloration and Bioengineering

**DOI:** 10.3390/biomimetics7020066

**Published:** 2022-05-23

**Authors:** Atrouli Chatterjee

**Affiliations:** Department of Cell Biology, Yale University, New Haven, CT 06520, USA; atrouli.chatterjee@yale.edu

**Keywords:** structural color, cephalopod, biophotonics, protein, optical materials

## Abstract

Most of us get inspired by and interact with the world around us based on visual cues such as the colors and patterns that we see. In nature, coloration takes three primary forms: pigmentary coloration, structural coloration, and bioluminescence. Typically, pigmentary and structural coloration are used by animals and plants for their survival; however, few organisms are able to capture the nearly instantaneous and visually astounding display that cephalopods (e.g., octopi, squid, and cuttlefish) exhibit. Notably, the structural coloration of these cephalopods critically relies on a unique family of proteins known as reflectins. As a result, there is growing interest in characterizing the structure and function of such optically-active proteins (e.g., reflectins) and to leverage these materials across a broad range of disciplines, including bioengineering. In this review, I begin by briefly introducing pigmentary and structural coloration in animals and plants as well as highlighting the extraordinary appearance-changing capabilities of cephalopods. Next, I outline recent advances in the characterization and utilization of reflectins for photonic technologies and and discuss general strategies and limitations for the structural and optical characterization of proteins. Finally, I explore future directions of study for optically-active proteins and their potential applications. Altogether, this review aims to bring together an interdisciplinary group of researchers who can resolve the fundamental questions regarding the structure, function, and self-assembly of optically-active protein-based materials.

## 1. Introduction

**Figure 1:**
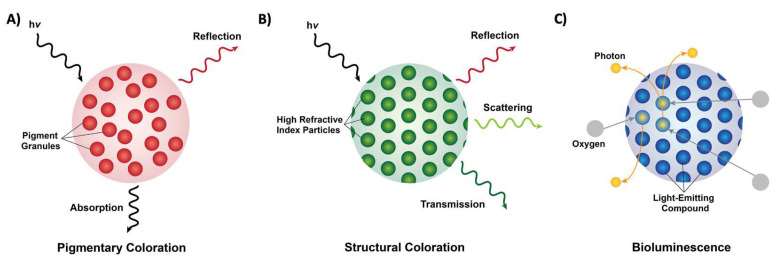
Forms of coloration in nature. (**A**) A schematic of pigmentary coloration in nature, where the incident light (h*v*) is absorbed or reflected by pigment granules (red circles), with no apparent long-range order. (**B**) A schematic of structural coloration in nature, where the incident light (h*v*) is reflected, scattered, or transmitted by high refractive index particles (green circles) showing long-range order. (**C**) A schematic of bioluminescence in nature, where oxygen (grey circles) reacts with a light-emitting compound such as luciferin (blue circles) and releases photons (yellow circles).

From the words on this page to the scenery outside, we as a species are extremely dependent on, and in many ways, inspired by the colors and patterns that we see around us. Coloration in nature can be categorized into three forms: pigmentary coloration, structural coloration, and bioluminescence (Figure 1) [1,2,3,4,5,6,7,8]. Pigmentary coloration is typically angle-independent coloration, where natural pigments directly absorb or reflect specific wavelengths of light (Figure 1A) [1,2,3,4,5,6,7,8]. Structural coloration is often angle-dependent and involves the interference (i.e., reflection, transmittance, or scattering) of light by biological micro- or nano-structures with contrasting refractive indices (Figure 1B) [1,2,3,4,5,6,7,8]. In comparison, bioluminescence typically involves the exergonic reactions of oxygen with different substrates and enzymes that lead to the production of visible photons of light (Figure 1C) [1,2,3,4,5,6,7,8]. The variety of colors that result from these three photonic mechanisms in nature serves numerous functions, including camouflage, mimicry, aposematism, signaling, photosynthesis, and self-protection [1,2,3,4,5,6,7,8]. These natural uses have inspired and led to the design and engineering of materials with dynamic color changing capabilities (e.g., for infrared camouflage), anti-counterfeiting technologies, and a variety of displays and sensors [9,10,11,12]. Nevertheless, the fundamental understanding of the molecular mechanisms that enable the formation (i.e., self-assembly) and dynamic tunability of these natural structures has been limited, which in turn has hindered the applications and engineering of biomolecule-based optical technologies. In this review, I showcase a few examples of pigmentary and structural coloration in nature and highlight cephalopods as model organisms for dynamic structural coloration. Next, I discuss the current state-of-the-art strategies for structural and optical characterization of proteins. Finally, I outline a few potential directions for the study and engineering of optically-active proteins. Overall, this review aims to bring together the collective fields of biophotonic materials and structural coloration to emphasize the need for collaboration and an interdisciplinary approach to shed light on the biochemistry behind these astounding molecules.

## 2. Pigmentary and Structural Coloration in Nature

Pigmentary coloration allows animals and plants to maintain bright coloration, which is often used to distinguish between species. Such forms of coloration are widely found in many mammals, birds, butterflies, fish and other marine organisms, and plants [13,14,15,16,17,18,19,20,21,22,23,24,25,26,27]. For example, mammalian skin and hair/fur typically contain melanin variants which give the animals their specific colorations [13,14]. Interestingly, pigments are also found in the eye, within retinal pigment epithelial (RPE) cells in humans, where melanin and lipofuscin serve to absorb light and thereby reduce light damage in the eye [15,16]. Similar to humans and other mammals, melanin is also a common pigment found in bird feathers and exposed structures (e.g., skin and legs) [17,18]. The colors of birds are particularly diverse because of their reliance on visual cues for communication [17,18]. Typically, birds produce both eumelanins (black, brown, and grey coloration) and pheomelanins (yellow and red coloration) via cells known as melanosomes, where coated vesicles are known to actively transport chemical precursors to induce the formation of different melanins [17,18]. In addition to melanins, birds are also known to utilize carotenoids (largely acquired via their diet), along with other pigments, including flavins and porphyrins [17,18]. On the other hand, butterflies, which also boast a wide variety of colors and patterns, have papiliochrome pigments (yellow coloration), the carotenoid lutein (blue–green coloration), and variants of these pigments depending on the species [19,20,21,22]. Notably, butterflies also use photoreceptors in their eyes to distinguish colors, likely in order to identify food sources, for mating, and to identify the locations of their host plants [23]. In contrast, fish have a variety of chromatophores (cells with pigment-based structures), including melanophores (which contain melanin), xanthophores (which contain yellow carotinoid-based pigments), and erythrophores (which contain red carotenoid-based pigments) [24,25]. More broadly, marine organisms also utilize carotenoids for their coloration, along with tetrapyrroles, quinones, azulenes, and melanins [26]. Some of the pigments found in these organisms are derived from their diets (e.g., carotenoids and tetrapyrroles), are found in the ink released by the organisms (e.g., tetrapyrroles in the purple ink of sea hares and melanin in the ink of cephalopods), or are produced by the organisms themselves (e.g., carotenoids in the lower trophic levels and anthraquinones in marine fungi) [26]. In comparison, the roles and diversity of pigments in plants likely surpass those known for animals, and specifically, plant pigmentation has a long history that is closely tied to the extraction and utilization of plant pigments as dyes and paints [27,28,29]. As one example, plants’ ability to perform photosynthesis is also critically dependent on pigment molecules such as chlorophylls (green pigments) and carotenoids (yellow, orange, and red pigments) [27,28,29]. Pigments such as flavonoids, including anthocyanins (pale yellow, pink/red, blue, and black pigments), and betalains (yellow or red pigments) can be found in the epidermal cells of flower petals, and are vital as visual signals for pollination and seed scattering, and thus in the interactions between plants and animals [27,28,29]. Altogether, pigments and pigment-based coloration represent a vibrant chemical modality for generating coloration; however, they generally produce angle-independent coloration that cannot be completely turned on or off, unlike structural coloration.

**Figure 2:**
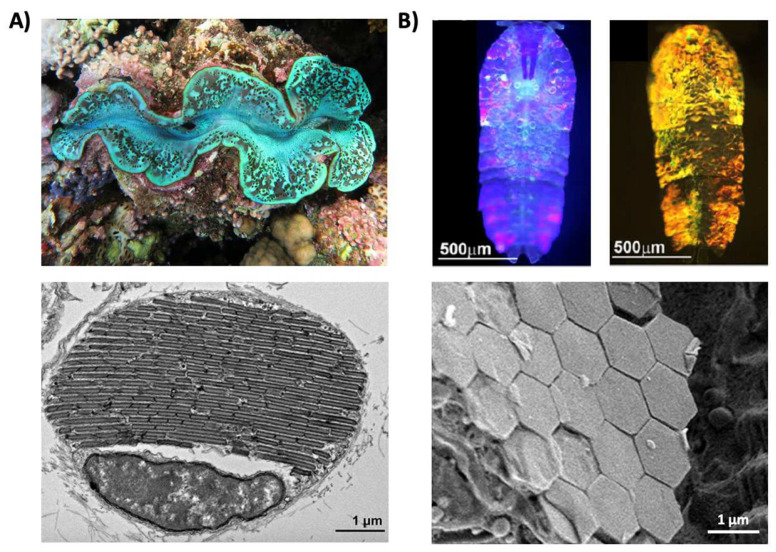
Structural color derived from guanine crystals. (**A**) (**Top**) An image of a giant clam (*Tridacninae*). (**Bottom**) An electron microscopy image of a giant clam iridocyte cell stacked with alternating layers of guanine crystal plates and cytoplasm sheets. (**B**) (**Top**) Images of male Sapphirinid copepods. (**Bottom**) A cryo-scanning electron microscopy image of an *S. metallina* copepod, showing hexagonal guanine crystals within iridophores below a chitin procuticle. The images in part (**A**) are reproduced from [30]. The images in part (**B**) are reproduced from [31].

**Figure 3:**
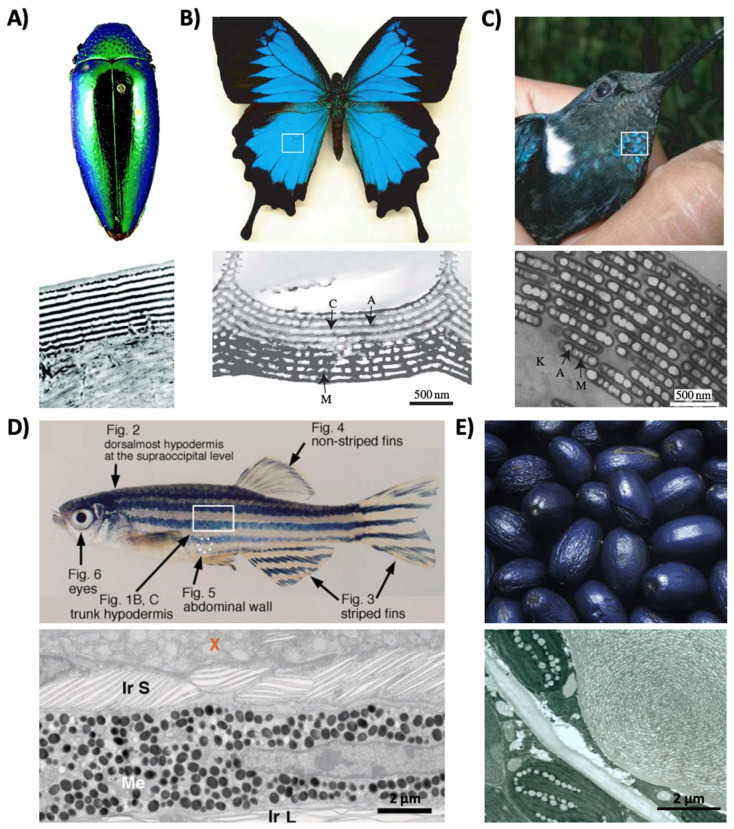
Structural color derived from 1D photonic crystal-like structures and iridophore cells. (**A**) (**Top**) An image of multilayer color in a buprestid or jewel beetle. (**Bottom**) An electron microscopy image of the cuticle of a *Cicindela scutellaris* beetle. (**B**) (**Top**) An image of a *P. ulysses* butterfly. (Bottom) A transmission electron microscopy image of the blue scale of the butterfly showing chitin (C) and air (A) arrays; the lower portion contains diffuse melanin (M). (**C**) (**Top**) An image of the *C. prunellei* hummingbird. (**Bottom**) A transmission microscopy image of a green barbule from a *C. iris* hummingbird, showing ordered layers of air (A) and melanin (M) in a keratin (K) matrix. (**D**) (**Top**) An image of a zebrafish. (**Bottom**) A transmission electron microscopy image of a portion of the zebrafish hypodermis showing xanthophores (X), type-S iridophores (Ir S), melanophores (Me), and type-L iridophores (Ir L). (**E**) (**Top**) An image of a *D. michieana* fruit. (**Bottom**) A transmission electron microscopy image of a transverse cross-section of a *D. michieana* fruit showing iridosomes. The images in part (**A**) are reproduced from [33]. The images in parts (**B**,**C**) are reproduced from [34]. The images in part (**D**) are reproduced from [35]. The images in part (**E**) are reproduced from [36].

Structural coloration is also found in a variety of organisms, but in comparison to pigmentary coloration, allows the organism to generate colors at very specific wavelengths depending on the geometry (sizes and order) of the structures formed. For example, guanine crystals are found in the iridocytes of giant clams (*Tridacninae*) and form hexagonal structures below the cuticles of Sapphirinid copepods (Figure 2), where the arrangement and geometries of the structures formed by the guanine crystals lead to very different spectra observed for the animals [30,31]. Other forms of structural colors in organisms include 1D photonic crystals, as observed in Japanese jewel beetles (*Chrysochroa fulgidissima*) and other buprestids, *Cicindela scutellaris* beetles, *Papilio ulysses* butterflies, and the *Coeligena prunellei* hummingbird (Figure 3A–C) [32,33,34]. These insects and birds have alternating layers of chitin/melanin, chitin/air, or air/melanin in a keratin matrix with different periodicities that result in either the metallic hues of the beetles or the blue–green colors observed in the butterflies and hummingbirds [32,33,34]. Other structures, such as the lamellar membrane morphology observed in the iridosomes of zebrafish and the *Delabrea michieana* fruit enable narrowband light reflectance or iridescence (Figure 3D,E) [35,36]. In general, the iridosomes reflect specific wavelengths of light with particulate structures formed from high-refractive-index materials arranged within ribbon-like structures that form a one dimensional photonic crystal-like architecture composed of alternating high and low refractive index areas in the cells [35,36]. Similar multilayer reflectors have also been observed in various other species of butterflies and fish [17,18,19,20,21,22]. Although these examples demonstrate the broad range of structures and thus examples of structural coloration, the colors themselves are typically static (i.e., unchanging or changing over relatively long time-scales) or are influenced by the viewing angle of the observer rather than controlled by the organism itself.

## 3. Cephalopods as Model Organisms for Dynamic Structural Coloration

**Figure 4:**
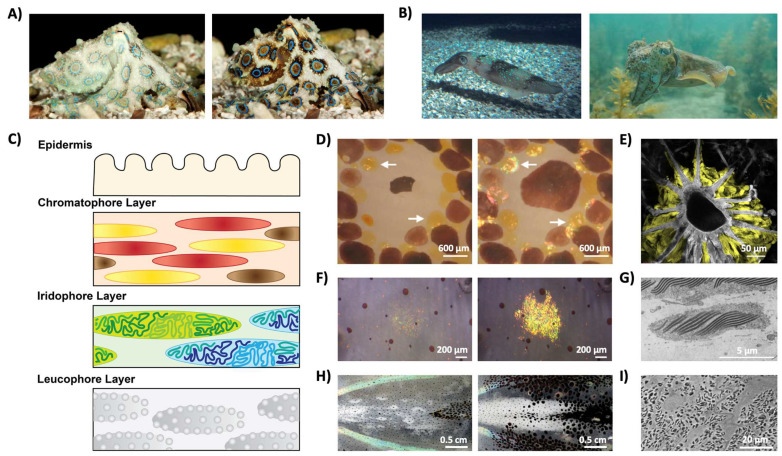
The color-changing abilities of cephalopods and their complex skin architectures. (**A**) Images of a blue-ringed octopus (*Hapalochlaena lunulata*) changing its coloration. (**B**) Images of a *Loligo pealeii* squid (**left**) and a *Sepia apama* cuttlefish (**right**). (**C**) A schematic of squid skin showing their complex skin architecture, which contains an outer epidermis and layers of chromatophore organs, iridophore cells, and leucophore cells. (**D**) The contracted (**left**) and expanded (**right**) forms of a chromatophore organ, where the white arrows show the presence of iridophores. (**E**) An electron microscopy image of a chromatophore organ, where the portions highlighted in yellow indicate the positions of sheath cells. (**F**) The non-iridescent (**left**) and iridescent (**right**) forms of an iridophore cell. (**G**) An electron microscopy image of an iridophore cell showing the intracellular lamellar structures (dark gray) that enable the narrowband reflectance characteristic of the cell. (**H**) The transparent (**left**) and opaque (**right**) tissue in the mantle of a female *Doryteuthis opalescens* squid. (**I**) An electron microscopy image of the layer of leucophores showing the electron dense proteinaceous particles called leucophores (dark gray) that enable the dynamic opacity-changing capabilities of the squid. The images in part (**A**) are reproduced from [36]. The images in part (**B**) are reproduced from [40]. The images in parts (**D**,**E**) are reproduced from [43]. The images in part (**F**) are reproduced from [46]. The image in part (**G**) is reproduced from [44]. The images in parts (**H**,**I**) are reproduced from [53].

Unlike most organisms known to date, coleoid cephalopods (i.e., squid, octopi, and cuttlefish) represent some of the very few examples of animals that are able to alter their appearances (both coloration and texture) almost instantaneously (Figure 4A,B) [37,38,39,40]. Specifically, the animals’ amazing color changing abilities are enabled by complex hierarchical skin architectures, which contain both pigment-containing organs and iridophore and leucophore cells, which act as narrowband and broadband reflectors, respectively (Figure 4C) [10,11,12,40,41]. The pigment-containing organs, called chromatophores, are shown in Figure 4D,E) [40,41,42,43]. They contain a central chromatosome cell (primarily filled with pigment granules) which is surrounded by enervated muscle cells that expand and contract to modulate the size of the pigment-filled cells, thereby allowing the chromatophore to act as a color filter (Figure 4D) [40,41,42,43]. Notably, researchers recently found that this organ also contains sheath cells, which have high-refractive-index protein particles that are thought to contribute to the structural coloration of the organ, although the precise biological function of the sheath cells remain unclear (Figure 4E) [43]. The iridophore cell, much like those seen in other organisms, enables narrowband light reflection via a Bragg-stack-like structure (Figure 4F,G) [44,45,46,47,48,49,50]. Briefly, a Bragg stack (or a distributed Bragg reflector) is a one-dimensional photonic crystal formed from alternating layers with different refractive indices, for which the intensity of reflected light, *R*, can be approximated by the following equation:

$$R={\left[\frac{n_{o}{\left(n_{2}\right)}^{2N}-n_{S}{\left(n_{1}\right)}^{2N}}{n_{o}{\left(n_{2}\right)}^{2N}-n_{S}{\left(n_{1}\right)}^{2N}}\right]}^{2}$$

where *n_o_* is the refractive index of the originating medium, *n*_1_ and *n*_2_ are the refractive indices of the alternating materials, *n_s_* is the refractive index of the substrate, and *N* is the number of repeated low/high-refractive-index pairs [50,51,52]. Note that this equation also assumes that all repeated low/high-refractive-index pairs have a thickness, *d*, equivalent to a quarter of the wavelength, or:

$$d=\frac{\lambda }{4n}$$

In cephalopod iridophores, the Bragg stacks are formed from alternating layers of a high-refractive-index protein called reflectin, and the extracellular space, which are separated by the cell membrane (Figure 4G and Table 1) [44,45,46,47,48,49,50]. Interestingly, in some cephalopods, these cells can respond to chemical stimulation and thereby alter the wavelength of the reflected light (Figure 4F) [44,45,46,47,48,49,50]. It is hypothesized that the chemical stimulation alters the aggregation of the reflectin proteins, thereby altering the relative spacing of the high-refractive-index layers of the Bragg stack and modulating the wavelength of the reflected light [44,45,46,47,48,49,50]. The leucophore cell functions to broadly scatter white light and can provide a bright white background to enhance the appearance of the colors produced by the chromatophore organs and the iridophore cell (Figure 4H,I) [10,11,12,40,42,53]. These cells typically contain high refractive index protein-based, membrane-enclosed granular structures, called leucosomes, that function to scatter light (Figure 4I and Table 1) [53]. In some squid, these cells are also dynamic and are able to reversibly change the appearance of the tissue layers from transparent to opaque with chemical stimulation (Figure 4H) [53]. Altogether, the combined capabilities of such cells and organs make cephalopods model organisms for the study of optically-active biological materials.

Within the context of cephalopod camouflage, reflectin proteins have gained significant research interest in the last several years. Reflectins are found in all of the cephalopod cells that enable structural coloration (e.g., sheath cells within the chromatophore organ, iridophore cells, and leucophore cells) [43,47,48,49,53,62]. The proteins in this family typically contain a large fraction of highly charged and aromatic residues, which gives them their unusually high refractive indices (see Table 1) [43,47,48,49,53,58,61,62]. In general, the proteins are composed of repeated subdomains connected with variable linker regions [58,62]. The subdomains vary for each isoform, but most isoforms contain the internal repeat motif, which has the following general sequence: PER-X_2_-DM-X_4_-MD-X_5_-MD-X_7_-P, where X represents sites with variable amino acids [58]. Interestingly, although the tertiary structure for full-length reflectins remains unknown (note that while theoretical work suggests the presence of secondary structural features, they have yet to be confirmed experimentally), recent work shows that the abundant internal motif has some secondary structural features which can be modulated with external stimulation [58]. Moreover, reflectins can be expressed in and purified from bacterial cells, and the resulting protein can self-assemble into a variety of structures, including nanoparticles, microfibers, hexagonal plates, and thin films [60,61,62,63,64,65,66,67,68,69]. Some of these structures can be further modulated by the application of external stimulation [58,60,61,63,65,66,67,68,69]. For example, mechanical agitation of nanoparticles has been shown to induce fibrillation for a truncated reflectin variant and the application of a unidirectional mechanical stimulus has been shown to alter the infrared reflectivity of thin films of reflectin [58,69]. Moreover, chemical stimulation can alter the aggregation state of nanoparticles (e.g., by altering the ionic strength of the solution), induce the assembly or disassembly of hexagonal plates (e.g., by introducing or removing aromatic compounds), and alter the relative thickness of thin films of reflectin (e.g., by altering the local humidity or pH) [60,61,63,66,67,68,69]. Notably, in mammalian cells engineered to produce intracellular reflectin structures, chemical stimulation induced the reorganization of the reflectin aggregates and was able to alter the relative opacity of the cells [60]. Altogether, the reflectin family of proteins represents versatile materials that show great potential for optical engineering.

To date, several strategies have been used for the fabrication of bioinspired photonic technologies. In general, inorganic materials have been processed via techniques such as two-photon polymerization, colloidal assembly strategies, and top-down lithography [70,71,72,73,74]. However, these techniques cannot be used on more-sensitive proteins, and instead, techniques such as doctor-blading and inkjet printing of thin films, the extrusion of fibers, and solvent-based self-assembly of particles in solution (e.g., organic solvent induced hexagonal plate formation) are more widely used [59,60,65,66,67,68,69,75]. Nevertheless, the development of reflectin-based biophotonic technologies has been largely limited to thin-film-based geometries partially due to the limited knowledge that we have so far of the proteins’ structure, self-assembly, and proclivity for forming aggregates under physiological conditions. More broadly, the development of protein-based engineering applications has been limited by the challenges associated with protein production, environmental sensitivity, and characterization. For example, the large-scale production of proteins while maintaining their native folding state is challenging, and as a result, many of the optical technologies engineered from proteins have remained at the laboratory-scale. As another example, most proteins are extremely sensitive to their local environment, which not only limits their application, but can also lead to degradation or material “defects” over time (for example, by the formation of aggregates or misfolded proteins) [76,77,78,79]. Moreover, proteins typically behave differently in vivo and in vitro, which means that reconstituting structures observed within the native organism in a solution is rarely straightforward. These issues, coupled with the ever-present challenges of characterizing protein structure and correlating the molecular structure with the physical properties of the protein, lead to a tremendous obstacle that, if resolved, could revolutionize not only our understanding of proteins, but would likely induce paradigm shifts across biology and materials engineering.

## 4. Techniques for the Structural and Optical Characterization of Proteins

Generally, in order to capitalize on the unique functional abilities of an optically-active protein, it is necessary to fully understand (a) the secondary, tertiary, and quaternary structural features of individual isoforms; (b) directly correlate the structure of the protein (either of a single molecule or a larger aggregate) with its optical properties (e.g., refractive index, polarizability); and (c) how multiple isoforms interact to form the optically-active structures in vivo. Currently, several technologies enable researchers to resolve the molecular structure of proteins, including Fourier transform infrared (FTIR) spectroscopy, Raman spectroscopy, circular dichroism (CD), X-ray diffraction (XRD) crystallography , and cryo-electron microscopy (cryo-EM). For example, FTIR spectra can provide fingerprints for proteins with predominantly alpha-helical or beta-sheet structures based on the vibrational energies of C=O, N–H, and C–N bond stretching and the separation between the amide absorption peaks; however, the peaks may become indistinguishable or demonstrate unexpected shifts in proteins with more than one secondary structural feature (which is often the case) [80,81,82]. In general, although the 3D structural information for a protein cannot be determined from this technique, FTIR spectroscopy is useful because there are no protein size limitations, it can be used on membrane proteins, and it also provides information on the local dynamic conformational changes of the protein [78,79,80]. Raman spectroscopy, like FTIR, measures the vibrational energies of amides to distinguish between alpha-helical and beta-sheet structures [83,84]. One advantage of Raman spectroscopy is that water appears relatively transparent due to the Raman effect, which simplifies spectral analysis, and as such the technique can also be used to identify disulfide bridges and aromatic amino acids (e.g., Phe, Tryp, Tyr, and His) [83,84]. Nevertheless, solvent, fluorescence, and temperature effects can make it challenging to deconvolute the secondary structures of proteins using Raman spectroscopy [83,84]. In comparison, CD uses differences in the phi and psi angles of the polypeptide backbone to identify relative fractions of alpha-helices, beta-sheets, turns, and random coils [65,66,67,68]. Recently, methods to cross-correlate simulated CD spectra from computationally simulated structures and experimental CD spectra have provided additional insight into the secondary and tertiary structures of proteins, and in general are excellent tools for the rapid determination of the secondary structures and folding properties of proteins [85,86,87,88]. XRD crystallography is a well-established tool for determining the 3D structures of proteins at nearly atomic resolutions by deciphering the diffraction patterns from protein crystals [89,90]. Proteins can be made to crystallize by precipitation under highly ionic conditions or by varying the concentrations of organic solvents, pH, temperature, or protein concentrations [86,87]. However, the technique has not been able to determine the 3D structures of all proteins, partially because not all proteins can be made to crystallize, and also because the induction of crystallization may also alter the native folding of the proteins [89,90]. As such, techniques that are able to characterize the native folding state of proteins without the need for crystals or “harsh” conditions, such as nuclear magnetic resonance (NMR) spectroscopy and cryo-EM, have been gaining popularity (note: as NMR is still only able to characterize the structures of peptides or relatively small proteins, the technique is not discussed here). Within this context, cryo-EM enables near-atomic-level (<4 å) structural determination of monodisperse macromolecular assemblies [91,92,93]. The assemblies are imaged at cryogenic temperatures in a transmission electron microscope, which along with recent computational advances, has streamlined the procedures for identifying and evaluating protein structures [91,92,93]. Altogether, the existing technologies can help researchers elucidate the structures of a variety of proteins; however, the size and stability of the protein often limits the resolution at which the molecular structure can be determined. Nevertheless, these strategies highlight the need for the improvements in methodologies for the prediction and evaluation of native protein conformations, particularly within the context of protein structure and optical function correlations.

Together with the challenges associated with protein structure determination, efforts to evaluate the native refractive indices of proteins have also been limited to date. In general, the refractive index of a material is a measure of the material’s ability to bend light and is mathematically expressed as the ratio between the speed of light traveling through the material to light’s speed through a vacuum. Although refractometry or ellipsometry is typically used to measure the refractive index of a material, because proteins are formed from the same component amino acids, their refractive indices are often assumed to be around 1.5 [94,95,96,97,98,99]. In addition to experimental methods, there are multiple theoretical methods with which to approximate the refractive indices of proteins, which utilize different assumptions and experimental datasets to derive a general rule for calculating the refractive indices of proteins [100,101,102,103,104,105,106]. Nevertheless, these methods (a) often use bulk refractive index measurements of amino acid solutions as the basis for their calculations (however, simple concatenation does not yield accurate refractive index values for proteins); (b) the correlations derived in the studies are often only accurate for specific proteins or specific classes of proteins (e.g., crystallins); and/or (c) the correlations are accurate under very specific conditions (e.g., for specific solvents, temperatures, or pH). Although new microscopic techniques, such as quantitative phase imaging methods, are becoming increasingly popular for the visualization of intracellular structures and in theory may be used to evaluate the refractive indices of intracellular structures, these techniques are inherently limited by the lack of standards that have the geometry (i.e., sizes) and refractivie indices required for precise optical characterization within the resolution limite of the instrument,, and instead rely on the statistics of the data collected by the user to evaluate the validity of the measurements [107,108,109,110]. As a result, there is a critical need for the development of strategies to fundamentally understand and to experimentally and theoretically evaluate the refractive indices of proteins.

## 5. Future Directions for the Study of Optically-Active Proteins

The need for the development of strategies to study and characterize both proteins’ structures and their enticing optical properties comes from the potential of these proteins in the design and engineering of a variety of unique classes of materials. For example, the determination of the structure–optical property correlation, along with an understanding of the molecular mechanism for the self-assembly of these proteins would provide general methodologies for engineering self-aggregating (or even self-crystallizing) protein structures, which would be revolutionary to the fields of protein engineering and materials design. Such structures could subsequently be used to guide the spatial propagation of light (e.g., protein-based waveguides), design bio-optical structures (e.g., artificial lenses), or even to modulate the optical signatures of larger structures, such as cells or tissues (e.g., increase the “transparency” of tissues to attain deep-tissue imaging modalities without the hassle or limitations of tissue clearing). Such designer protein structures or protein-containing cells could have several uses in medicine or biomedical engineering, including as natural markers for diagnostics or intracellular labeling (e.g., the utilization of a protein’s high refractive index as a phase contrast agent using similar principles and established fluorescence markers), artificial materials for optical procedures (e.g., artificial corneal matter with designer optical capabilities), or biological diagnostic/prognostic tools with protein-based optical sensors (e.g., a device that can be embedded within the body that changes color by changing the organization of a protein matrix). In fact, the possibilities for utilizing proteins with tunable optical properties are limitless and are only confined by our understanding of the materials.

Within this context, the field of protein-based biophotonic materials critically requires the insight of biochemists that are well-versed in techniques to probe and determine both the molecular structures of the proteins and the biochemical mechanisms by which the optically-active structures are formed. In particular, strategies such as cryo-electron tomography (cryo-ET), which combines the resolution of electron microscopy with the multi-angle resolution of tomography on a cryogenically frozen biological sample, can offer new structural information of large molecular assemblies [111,112,113,114,115]. Recently, cryo-ET has been used successfully to study membrane fusion in viral systems; to understand intracellular processes, such as the arrangement of the molecular machinery for vesicle docking, fusion, and release; and the molecular organization within neuronal cells [114,115]. Furthermore, the technique may be promising for the structural characterization of proteins such as reflectins that are too small for cryo-EM studies and do not crystallize, but which readily form aggregates in solution. Moreover, biochemists have been working for decades on reconstitution strategies that enable them to mimic native processes and thereby evaluate the mechanistic and kinetic pathways (such as the vesicle fusion pathways) which are ubiquitous in most animals. These strategies, which rely on the preparation of physiological buffer conditions, the identification of the minimal biomolecular machinery and the relative composition necessary for the processes, and evaluation of the precise amino acid residues necessary for the native functionality (e.g., via mutation studies), would allow researchers in the field of protein-based optical materials to identify the molecular mechanisms that enable these dynamic proteins to organize themselves into the structures of interest. In particular, within the context of cephalopod protein-based dynamic coloration, kinetics-based biochemical approaches could provide insight into the molecular mechanism by which protein reconfiguration at the intracellular level and chemical actuation at the cellular level enable the rapid color changing capabilities of the organisms. In parallel, the methodologies developed for the characterization of the structures of optically-active proteins will also support the improvement of general strategies for protein structure determination, thereby also supporting the technological advancement of tools used by biochemists. Moreover, the elucidation of the self-assembly methods for optically-active proteins could also provide new insight into the general pathway by which protein aggregates form, which would have significant implications for a variety of neurological diseases. For example, neurological disorders such as Alzheimer’s disease, Parkinson’s disease, Huntington’s disease, and amyotrophic lateral sclerosis (ALS) are generally thought to be caused by the formation of protein aggregates that consist of misfolded proteins; however, there is limited understanding of (a) the molecular mechanism by which such aggregates are formed and (b) the mechanisms that inhibit the natural degradation of the misfolded proteins [116,117,118,119,120]. Optically-active proteins, such as reflectins, typically contain a large fraction of charged and aromatic residues, which make them prone to forming aggregates under physiological conditions. The utilization of such proteins as markers or the in vitro characterization of the optically-active proteins’ ability to self-assemble could provide new insight into the biochemistry of the onset of such neurological diseases. Taken together, there can be no doubt that to truly understand this class of optically-active proteins, an interdisciplinary approach must be taken.

## 6. Conclusions

Altogether, nature presents many interesting optically-dynamic structures, which continue to serve as templates and as sources of inspiration for bioengineers. Among the diverse array of structures formed within the wings, shells, feathers, furs, scales, and skin of a variety of organisms, the complex cellular architecture of cephalopod skin is of particular interest because of the multifaceted role that reflectins play in forming the dynamic optically-active cells that generate structural color for octopi, cuttlefish, and squid. Although several methods currently exist to study and characterize the structures and optical properties of these proteins, there still remains no direct method for determining the structure, optical properties, and their correlation for any family of proteins. As a result, this review aims to connect the fields of optical materials design and engineering, structural coloration, and biochemistry who may be able to uncover the properties of these unique biomolecules.

## Figures and Tables

**Table 1:** The refractive indices of optically-active biomolecules.

Protein	Refractive Index	Description	References
Melanin	1.7–1.8	Bird of paradise melanin measured with Jamin-Lebedeff interference microscopy fit with the Cauchy equation	[54]
Keratin	1.532	Bird (*Anas anas domestica*) keratin measured with Jamin-Lebedeff interference microscopy fit with the Cauchy equation	[55]
	1.54–1.57	Bird of paradise keratin measured with Jamin-Lebedeff interference microscopy fit with the Cauchy equation	[54]
Chitin	1.517	Butterfly (*Graphium Sarpedon*) chitin measured with Jamin-Lebedeff interference microscopy fit with the Cauchy equation	[55]
Reflectin	1.41	Cuttlefish leucosomes	[56]
	1.44	Condensed platelets in squid iridophores	[57]
	1.40–1.47	Structures formed in solution of a truncated reflectin variant	[58]
	1.54–1.59	Reflectin-based substrates	[59,60]
	1.42–1.62	Reflectin-based structures in engineered cells	[61]
